# Photosensitivity and Carrier Densities of Perovskite Solar Absorbers

**DOI:** 10.1002/advs.202412711

**Published:** 2025-02-25

**Authors:** Małgorzata Kot, Katarzyna Gawlińska‐Nęcek, Emilia Pożarowska, Karsten Henkel, Dieter Schmeißer

**Affiliations:** ^1^ Institute of Physics Brandenburg University of Technology Cottbus‐Senftenberg Konrad‐Zuse‐Straße 1 03046 Cottbus Germany; ^2^ Faculty of Electronics Photonics and Microsystems Wroclaw University of Science and Technology Janiszewskiego 11/17 Wroclaw 50–372 Poland; ^3^ Institute of Metallurgy and Materials Science Polish Academy of Sciences Reymonta 25 St. Krakow 30059 Poland

**Keywords:** bipolarons, ionicity factor, large polarons, perovskite solar cells

## Abstract

Dark and light current–voltage characteristics of perovskite solar absorbers are analyzed in terms of their carrier densities. The analysis reveals p‐type large polarons as a dominant carrier type in the investigated perovskite solar cells. The mechanism causing photosensitivity is attributed to the dissociation (and pairing) of bipolarons to large polarons (and vice versa) that are controlled by the internal potential Γ. As an example, the polaron concept is tested for a formamidinium lead triiodide perovskite solar cell. The individual steps of the data analysis are demonstrated and determine the ionicity factor of this perovskite film, quantify the density of the large polarons, and predict the gain and loss of photo‐induced carriers. It is deduced that a reversible light‐on/off operation can only occur when the bias voltage never exceeds a critical value of the internal potential. The results gained in this study suggest that the novel analysis can be successively applied on different hybrid perovskite materials, too.

## Graphical abstract

A novel analysis of the dark and light *I–V* curves of perovskite solar cells enables the determination of the charge carrier density, the extraction of the ionicity factor, and the quantification of the gain and loss processes, to name a few. The mechanism causing photosensitivity is attributed to the dissociation/pairing of bipolarons/p‐type large polarons controlled by the internal potential.

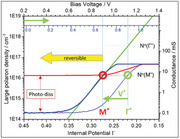

## 1 Introduction

Perovskite solar cells (PSCs) have gained significant attention in recent years due to their impressive power conversion efficiencies (>26%) and their potential for low‐cost and high‐performance photovoltaic devices.^[^ ^1^ ^]^ Current research activities include: enhancement of the open circuit voltage (V_oc_) values, improvement of PSCs long‐term stabilities, and optimization of large‐scale processing technologies.^[^ ^2^, ^3^, ^4^, ^5^, ^6^, ^7^, ^8^, ^9^, ^10^, ^11^ ^]^ Still, the materials parameters and mechanisms required to optimize their overall device performance and their efficiency are debated in literature.^[^ ^12^, ^13^ ^]^ In particular, limiting factors and loss mechanisms are an open field in materials research.^[^ ^14^, ^15^, ^16^, ^17^ ^]^

Recently, the properties of perovskite materials have been correlated via the concept of polarons.^[^ ^18^, ^19^, ^20^, ^21^, ^22^, ^23^ ^]^ In particular, the formation of large and small polarons (LaPs, SmaPs, respectively) in hybrid perovskites is considered. The coexistence of more covalent LaPs and more ionic SmaPs points to a mixed covalent–ionic system in which the large number of LaPs indicates a reduced iconicity of the material. The LaPs are multi‐atomic quasi‐particles, which are stabilized by covalent interactions; they are strictly separated from the positive counter charges that stay localized as SmaPs within the occupied iodine 4p/lead 5p valence states. Their spatial dimension exceeds several bond lengths making recombination processes very unlikely. They represent the mobile carriers and they carry a charge that is screened by their multi‐atomic polarization cloud. The carrier dynamics can be described in the context of polaron – bipolaron (P‐BiP) equilibria.^[^ ^23^, ^24^, ^25^, ^26^, ^27^, ^28^ ^]^ This scenario^[^ ^18^, ^19^, ^20^, ^21^, ^22^, ^23^, ^24^, ^25^, ^26^, ^27^ ^]^ considers covalent interactions among the intrinsic defect states within the ionic gap that are much stronger than next‐neighbor interactions via lattice distortions and this contrasts numerous concepts with more localized polarons.^[^ ^29^ ^]^

We employed the large polaron concept^[^ ^18^, ^19^, ^20^, ^21^, ^22^, ^23^, ^24^, ^25^, ^26^, ^27^ ^]^ to develop a model to identify quantitatively the role of LaPs and BiPs in the photosensitivity and the rectifying properties of gallium oxide (Ga_2_O_3_).^[^ ^28^, ^30^, ^31^ ^]^ There the mechanisms have been attributed to the pairing and dissociation of LaPs and BiPs by internal dipole momenta and this concept is applied here for perovskite systems, too. Here, the presence of LaPs is deduced from the continuous density of intrinsic defect states within the ionic bandgap as detected by resonant X‐ray photoelectron spectroscopy (resPES) studies.^[^ ^28^, ^32^ ^]^ The energetic width and position of these defects throughout the ionic gap suggest that the LaPs are band‐like carriers and this is the basis for our approach.

In this paper, we describe the analysis of the current–voltage (*I–V*) characteristics and we apply it for formamidinium lead triiodide (FAPbI_3,_ shortly FAPI) perovskite. The data are acquired in both, dark and light conditions, whereas the former one has often been omitted in the literature to date. This analysis of the *I–V* characteristics reveals the p‐type P^1π^ LaPs as the dominant carrier type. The mechanism causing photosensitivity is highlighted and attributed to the pairing of P^1π^ LaPs and dissociation of B^2π^ BiPs as controlled by contributions to the internal potential Γ. We are able to determine the ionicity factor of the perovskite film, to quantify the number of LaPs, to predict the gain and/or loss of photo‐induced carriers.

## 2 Experimental Section

### 2.1 Materials

**Figure 1:**
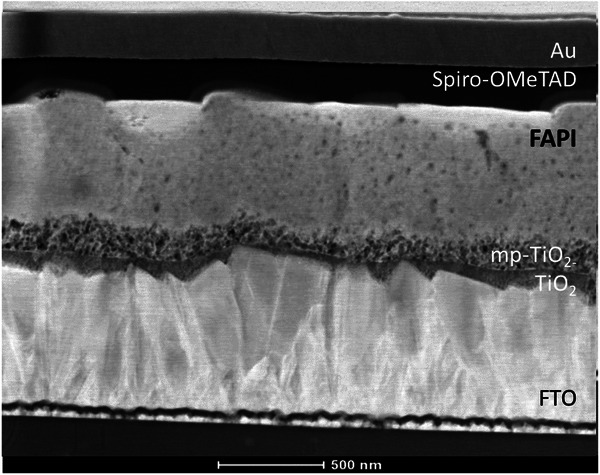
Cross‐sectional TEM image of the investigated FAPI‐based perovskite solar cell.

The FAPI‐based perovskite solar cells (see **Figure** **1**) were prepared on a fluorine‐doped tin oxide (FTO) glass. The blocking titanium dioxide (TiO_2_) layer, a thin TiO_2_ layer, and a framework of mesoporous (mp) titanium oxide are spin‐coated on top. The FAPI perovskite layer was spin‐coated on this FTO/TiO_2_/mp‐TiO_2_ substrate and heated to ensure the formation of the black‐colored FAPI phase. A 2,2′,7,7′‐Tetrakis[N,N‐di(4‐methoxyphenyl)amino]‐9,9′‐spirobifluorene (Spiro‐OMeTAD) film was used as a hole‐transporting layer. The whole process was completed by the deposition of a gold electrode. Further details of the preparation procedure are described in the Supporting Information file and in ref. [33]

### 2.2 Methods

#### 2.2.1 Cross section Transmission Electron Microscopy (TEM)

The cross‐section of the prepared FAPI‐based PSCs was investigated by using a TECNAI G2 F20‐200 kV FEG transmission electron microscope (TEM) in the bright field mode (BF). As shown in Figure 1, the perovskite layer is continuous with fine grains. The thickness of the perovskite film is ≈750 nm. It is sandwiched between the TiO_2_ blocking layer (20 nm) with porous scaffold (30 nm), the Spiro‐OMeTAD (140 nm), and the gold electrode.

#### 2.2.2 Current–Voltage (*I–V*) Measurement

Photovoltaic performance of the FAPI‐based PSCs was monitored by *I–V* characteristic using a Keithley 2401 source meter under simulated AM1.5G irradiation (100 mW cm^−2^). A Photo Emission Tech AAA class solar simulator was calibrated against certified reference silicon (Si) solar cells with a KG‐3 filter (Institute Fraunhofer ISE, Breisgau, Germany). The illuminated area of the solar cell was masked to 0.25 cm^2^. For the here presented analysis, we used the corresponding *I–V* curves that were performed in two scan directions, in forward (from −2.0 to 2.0 V) and reverse (from 2.0 to −2.0 V) bias mode with a scan rate of 200 mV s^−1^.

## 3 Results

**Figure 2:**
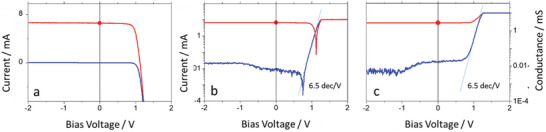
The dark (blue) and light (red) *I–V* data of the FAPI device in forward direction: a) linear scale, the red dot marks the value of I_sc_ = 6.6 mA at V_bias_ = 0 V; b) the data in logarithmic presentation; c) represented as conductance data. In (b) and (c) the exponential slope of 6.5 dec V^−1^ is indicated by the thin blue line.

In **Figure** **2**a, we show the *I–V* curves of the above‐mentioned FAPI perovskite solar absorber under dark and light conditions. We used a pristine sample in order to avoid contributions of possible degradation effects caused by previous characterization and storage. The data are taken within the bias voltage (V_bias_) range from −2.0 to +2.0 V and we would like to emphasize that both, the dark (blue) and the light (red) curves give a stable performance starting from V_bias_ = −2 V. For the here presented analysis we used the corresponding *I–V* curves that were performed in two scan directions, in forward (from −2.0 to 2.0 V) and reverse (from 2.0 to −2.0 V) bias mode with a scan rate of 200 mV s^−1^. A common analysis of the *I–V* data (see Figure S1, Supporting Information) of this device revealed the following parameters: an efficiency of 22.15% with a short circuit current (density) I_sc_ = 6.6 mA cm^−2^ (J_sc_ = 26.2 mA), an open circuit voltage V_oc_ = 1.12 V, and a fill factor FF = 75% with the maximum power point of 5.55 mW at a current of 6.1 mA and a voltage of 0.91V.

To analyze the *I–V* characteristics we use only the data of the first forward scan. We attribute the minor changes that occur in the following reversed scan to the stress applied to the PSCs by scanning up to +2.0 V thereby leaving the reversible regime (see below). Remarkable is that the current of the light curve appears rather stable along a wide bias voltage range with a value of ≈6.6 mA (red dot at V_bias_ = 0 V). This is also evident in the logarithmic presentation of these data in Figure 2b. Next, we convert the original data of FAPI (Figure 2a) to the conductance versus voltage (*G‐V*) representation by multiplying the (dark and light) current values by ((V_bias_ /A)–1)^−1^ and adding an offset G^0^. Important is to note that this procedure works for any value of the parameters A (in V) and G^0^ (in mS) and maintains the slope of the dark curve. We used values of A>50 V and adjusted G^0^ to obtain the saturation value of the conductance at 6.6 mA V^−1^ (red dot in Figure 2c at V_bias_ = 0 V). Thereby, the data now appear similar to the logarithmic *I–V* presentation of Figure 2b. In particular, the two characteristic features, the exponential V_bias_ dependence of the dark curve and the V_bias_ independent constant current value of the light curve remain unchanged while the zero‐voltage crossings are removed. In addition, the details of the dark curve become prominent and show an almost constant (but rumpling) lowest value in the regime from −2 V up to about V_bias_ ≈ +1 V that is followed by a pronounced increase in intensity and the exponential V_bias_ dependence with a slope of 6.5 dec V^−1^ in the range between +0.6 and ≈+1.1 V. The intensity of the dark curve increases by illumination to the saturation value of 6.6 mS and the light curve appears constant from V_bias_ = −2.0 to V_bias_ ≈ +1.0 V.

**Figure 3:**
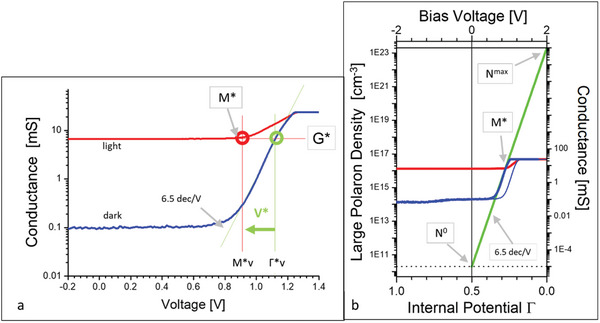
a) The detailed graphical analysis of the *G–V* data of FAPI based solar cells is shown previously in Figure 2c. It contains the exponential slope, the conductance value G^*^, and of the characteristic potentials Γ^*^v and M^*^v as well as the dipole V^*^. b) Presentation of N^π^(Γ)[P^1π^] between N^0^ and N^max^ as a function of the internal potential Γ, the measured dark curve (thin blue) is shifted by V^*^ (see below) to merge with the exponential slope of the p‐type P^1π^ LaPs (green) and with the M^*^ point of the light curve (red).

The logarithmic presentation of the *G–V* data of the FAPI device in **Figure** **3**b provide an intermediate step for the determination of the carrier densities. For that particular analysis, we need to introduce a dimensionless and normalized internal potential Γ^[^ ^31^ ^]^ of the sample as:

(1) $$\Gamma =\left[\left(\mathrm{VL}-V^{\pi }\right)-V^{0}+V_{\mathrm{bias}}\right]/E_{\mathrm{ionicgap}}$$

Here, the value of Γ reflects its position within the ionic gap (E_ionicgap_). It depends on the applied bias potential V_bias_. It also depends on the sum of all internal dipoles V^π^ = V* + V^pair^ + V^light^ caused by internal losses (V*), pairing equilibria (V^pair^), or by light (V^light^), as explained below.

We consider that the ionic gap of FAPI is filled with the intrinsic defect states (IDS) that are localized by Coulomb forces but with their covalent contribution the charges become de‐localized over several unit cells and some of them become mobile.^[^ ^31^ ^]^ The electronic structure of the lead‐iodide perovskites is deduced from our earlier resPES data analysis.^[^ ^32^ ^]^ The main features are the width of the ionic gap of 4 eV, the ionization potential V_L_, the electron affinity level V_U_, and the midgap position V^0^. Correspondingly, the internal potential Γ (with values between 1 and 0) spans the voltage regime from −2 V up to +2 V, respectively. Based on resPES data from several perovskite materials, in our analysis, we consider the ionic gap (pure Coulomb interactions) with the referred energy values to be similar in the family of the lead‐iodide perovskites. In contrast, the covalent contributions will be sensitive to variations of the counter ions, as these influence the energetic distribution of the IDS. Then, the ionicity factor determines their concentration (relative to the number of all valence electrons)^[^ ^34^ ^]^ and their energetic distribution determines the optical transitions, the absorption, and luminescence properties.

Next, the bias voltage (top scale in Figure 3b) is expressed by the internal potential Γ (bottom scale in Figure 3b) that controls the carrier density N^π^(Γ) of the mobile p‐type LaPs [P^1π^] and that can be calculated by:

(2) $$\begin{array}{ccc} N^{\pi }\left(\Gamma \right)\left[P^{1\pi }\right] & = & N^{\min}\cdot {\left[\exp\left\{\left(\left(1-\Gamma \right)\cdot E_{\mathrm{ionicgap}}\right)/\mathrm{kT}\right\}\right]}^{\left(1-\mathrm{fi}\right)}\\ & = & N^{\min}\cdot \exp\left\{\left(1-f_{i}\right)\cdot \left(\left(1-\Gamma \right)\cdot E_{\mathrm{ionicgap}}\right)/\mathrm{kT}\right\} \end{array}$$

where N^max^ = N^min^⋅exp{(1‐fi)⋅E_ionicgap_/kT}.^[^ ^31^ ^]^

This description is based on a single type of carriers, their density N^π^ depends on the ionicity factor f_i_ and on the internal potential Γ. This includes the photo‐induced increase of the carriers from the dark curve to the light curve. The p‐type LaPs reside in the BiP‐band within the ionic gap (see Figure S2, Supporting Information) and are stabilized by the covalent interactions and their population is a fraction of the intrinsic defect states within the ionic gap. In a recent study, we successfully demonstrated the applicability of this model to describe the photo‐sensitive properties of different mixed covalent‐ionic systems (different ionicity and ionic gap).^[^ ^28^ ^]^

The analysis of the *G–V* data of Figure 2 also indicates (in the context of Equation (2)) that FAPI is a mixed ionic‐covalent system. This is deduced from behavior of the intensity of the light curve that stays constant over a wide range of bias from V_bias_ = –2.0 V to the value of the characteristic potential M^*^. This represents a combined ohmic‐exponential behavior and the dark and the light curves start with the ohmic range always at Γ = 1 (V_bias_ = −2 V) and the respective value remains constant up to the intersection with the exponential N^π^(Γ) (green line in Figure 3b). Equation 2 represents the mobile charge carriers in perovskites, and it is used to explain the dark and light *G–V* characteristics of the FAPI device (Figure 2c and Figure 3a).

Further, in the curves in Figure 2c and Figure 3a we identify two points Γ^*^(= 0.22, the corresponding Γ^*^_V_ equals 1.12 V) and M^*^(= 0.27, respectively M^*^_V_ = 0.92 V). Please note that the parameters indicated with only ^*^ are related to the dimensionless internal potential Γ units while the parameters indicated with ^*^_V_ are related to the V_bias_ scale. Γ^*^ marks the intersection of the (extended) horizontal constant conductance value (G^*^) with the exponential slope of the dark curve and the M^*^ appears at the end of the constant ohmic G^*^ value of the light curve. The difference between Γ^*^ and M^*^ suggests that there is a shift of the dark curve with respect to the light curve as the M^*^_V_ value appears separated by V^*^_V_ = 0.2 V (V^*^ = 0.05). Note that this shift is blurred in Figure 2a,b by the zero crossings and it is not discussed or explained in the literature yet. Our analysis in Figure 3b enables us to correct for that difference of V^*^_V_ by shifting the dark (thin blue) curve and now the resulting bold blue curve intersects with the light (red) curve in the M^*^(M^*^_V_ = 0.92 V) point (see also ref.[28]). In **Table** **1** we collect all the quantities derived from the analysis shown in Figure 3.

**Table 1:** The most important parameters (ionicity factor f_i_ and the corresponding covalent fraction (1‐f_i_), internal potential M^*^(M^*^_V_) and Γ^*^ (Γ^*^_V_), loss dipole V^*^(V^*^_V_), carrier densities of N^0^, N^π^(M^*^), and N^π^(Γ^*^)) derived in this work from the analysis of the dark and light *I–V* characteristics of the corresponding solar cells with FAPI as the active material.

Parameter	f_i_	1‐f_i_	M^*^ [M^*^_V_]	V^*^ = M^*^‐Γ^*^ [V^*^_V_]	Γ^*^ [Γ^*^_V_]	N^0^ /cm^−3^	N^π^[M^*^] /cm^−3^	N^π^[Γ^*^] /cm^−3^
FAPbI_3_	0.62	0.38	0.27 (0.92 V)	0.05 (0.20 V)	0.22 (1.12 V)	1.2⋅10^10^	1.4⋅10^16^	3.0⋅10^17^

## 4 Discussion

The following sections give the basis for a quantitative discussion of loss and gain processes of the FAPI device. The *G‐V* data in Figure 2c and Figure 3a are free of zero crossings and they enable the determination of the ionicity factor f_i_ and of the LaPs density is based on the synergies between the dark curve of the *G–V* data and a quantitative analysis (Equation (2)) of the mobile LaPs carrier densities N^π^(Γ). This direct linkage shown in Figure 3b is highlighted by the direct analogy of the exponential slope of 6.5 dec V^−1^ found in the conductance data in Figure 2c and Figure 3 and in the logarithmic presentation (Figure 2b).

In Figure 3b the top and the bottom x‐axes are linked by Equation.(1) for V^π^ = 0. Note, that an increase of Γ is equivalent to a decrease of V_bias_. The main advantage, however, is given by the linkage of the y‐axes as the left‐hand axis N^π^(Γ) is quantitative by the value of N^max^ (= N^π^(Γ = 0)) = (1‐f_i_)⋅N^valence[^ ^31^ ^]^ that directly relates to N^valence^ = 5⋅10^23^cm^−3^, the fundamental value of the perovskite family. The fixed points at N^0^ (Γ = 0.5) and N^max^ (Γ = 0) are displayed in Figure 3b as horizontal black lines and are connected by the green exponential line. Consequently, the value of N^min^ is related to Γ = 1.

**Figure 4:**
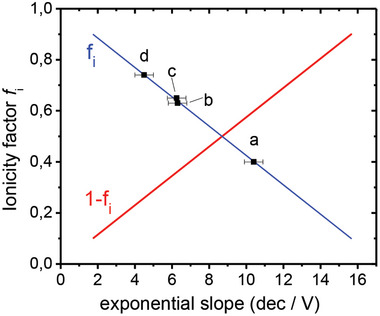
The relation of exponential slope of the dark curves with the ionicity factors f_i_ (blue) and the corresponding covalent fraction (1‐f_i_) (red). The data points are derived from the data of Figure 2 (b, FAPI), and also from other perovskite samples: CsMAFAPbI_3_^[^ ^7^ ^]^ (a), MAPbI_3_^[^ ^12^ ^]^ (c), and MAFAPbI_3_^[^ ^3^ ^]^ (d); the error bars indicate the variations of the dark curve slopes of the different samples of the same series, whose value is mainly based on the graphical analysis.

The ionicity factor of the FAPI device can be extracted with the frame of Figure 3b from the slope of the green curve (Equation 2). Starting from N^max^ the N^0^(@V_bias_ = 0 V)) value (dotted horizontal line) is lowered by ≈13 decades. Thus, the experimental *G‐V* slope of the dark curve of 6.5 dec/V is translated to values of N^max^(@V_bias_ = +2 V) = 2⋅10^23^cm^−3^ and N^0^ = 1.2⋅10^10^cm^−3^ (N^min^ = 2⋅10^−3^ cm^−3^). These values correspond (Equation (2)) to the ionicity factor f_i_ = 0.62 with a covalent fraction of (1‐f_i_) = 0.38 (see **Figure** **4** and Table 1). These values are obtained by applying Equation (2) to remove the small uncertainty by the initially guessed value of (1‐f_i_) in N^max^.

The analogy between the conductance ((*G–V*), right and top axes) and the carrier density N^π^[P^1π^](Γ) (left and bottom axes) is obtained by adjusting the experimental dark and light curves in such a way that at the M^*^ point they merge with the exponential N^π^[P^1π^](Γ) dependence (green curve). Thereby, the quantitative analysis of the LaPs densities N^π^[P^1π^](Γ) of any value of Γ is enabled. This direct relation combines two different physical quantities without the knowledge of the mobility, film thickness, and electrical field strength. It represents a novel analysis method that contrasts the more complex differential charging techniques^[^ ^10^, ^11^, ^28^, ^35^ ^]^ as it is free of any other parameter and is solely based on the experimental *G–V* data.

As an example, the light curve (red line in Figure 3) of the FAPI device with its conductance value of G^*^ is linked via the M^*^ = 0.27 point to the P^1π^ carrier density N^π^(M^*^) = 1.4⋅10^16^cm^−3^ (Figure 3b). Note, that this N^π^ value is independent on any normalization procedures of the *G–V* data due to the fact that both dark and light curve are shifted by G^0^ (i.e., in parallel to each other). In particular, it works also for analysis of the current densities. The reason is that only the data are shifted on the N^π^ axis until the slope of the dark curve coincides with that of the N^π^(Γ) curve at the M^*^ point – only the logarithmic relation between dark and light curves has to be maintained. This value of the photo‐dissociated P^1π^ LaPs is the first reported value of a carrier density of an illuminated perovskite film, to our knowledge.

In Figure 4 we plot the f_i_‐value (here, 0.62) against the value of the observed experimental slope (6.5 dec V^−1^) as derived from the analyzed data (Figures 2 and 3). We plotted also the results of our analysis to other perovskites such as MAFAPbI_3_,^[^ ^3^ ^]^ MAPbI_3_,^[^ ^12^ ^]^ and CsMAFAPbI_3_^[^ ^7^ ^]^ (manuscript in preparation), where we identified different slopes in the *G–V* characteristics ranging between 4 dec V^−1[^ ^3^ ^]^ and 11 dec V^−1^.^[^ ^7^ ^]^ All experimental *I–V* data of the above‐mentioned perovskites show the mixed ohmic‐exponential behavior of the dark curve (starting with a constant (ohmic) value and changing to an exponential dependence); this is the characteristic feature of mixed covalent‐ionic systems with a fractional f_i_ value. This comparison demonstrates that the exponential slope (as summarized in Figure 4) is a characteristic feature of perovskite films and devices.

The ionicity factor f_i_ gives the fraction of covalent intrinsic defect states to the total number of valence states N^valence^. Its exponential dependence on Γ requires that any dipole contribution to the value of Γ causes a change of P^1π^ LaPs according to δN^π^[P^1π^]/δΓ = 6.5 dec V^−1^ or 1 dec/154 mV for investigated FAPI device. Further, the dimension (1‐f_i_) of the exponential in Equation (2) reflects the collective nature of the LaPs. It underlines the multi‐atomic nature of the LaPs quasi‐particles^[^ ^18^, ^19^, ^20^, ^21^, ^22^, ^23^, ^34^ ^]^ as it indicates that their charge is stabilized within a multi‐atomic polarization cloud with a spatial dimension of several (1/(1‐f_i_)) neighboring sites. Thus, the fractional f_i_ value represents the evidence for intrinsic defect states (IDS) within the ionic gap, the existence and dimension of the LaPs, and their exponential dependence on the internal potential. These important details of the perovskite systems are directly evident in the experimental dark curves pointing to the importance of analyzing both, the dark and the light curves, indeed.

Now we relate the specific carrier densities N^π^[P^1π^](Γ) of FAPI in detail to the characteristic experimental values: the slope, the critical points Γ^*^ and M^*^, and discuss quantitatively the individual contributions of Γ to the carrier density N^π^(Γ). In the data of the FAPI device (Figure 3b) a value of Γ^*^ = 0.22 is found and it marks the lowest possible value for the non‐compensated P^1π^ density at N^π^(1‐Γ^*^ = 0.78) = 10^3^ cm^−3^ and the upper limit of N^π^[P^1π^](Γ^*^ = 0.22) = 3 ⋅10^17^cm^−3^. According to Equation (2) at Γ = 0 the population of P^1π^ LaPs could reach values up to N^max^ = 2⋅10^23^cm^−3^ if only the P^1π^ LaPs would exist. However, we have to consider that the dipole V^*^ in real perovskite systems describes the loss mechanisms caused by charge fluctuations within the complete IDS system in the ionic gap. They sum up, form oriented dipoles, and limit the carrier densities further as their sum adds as V^*^ to the actual value of Γ (V^π^ in Equation (1)) (see also ref.[28]).

**Figure 5:**
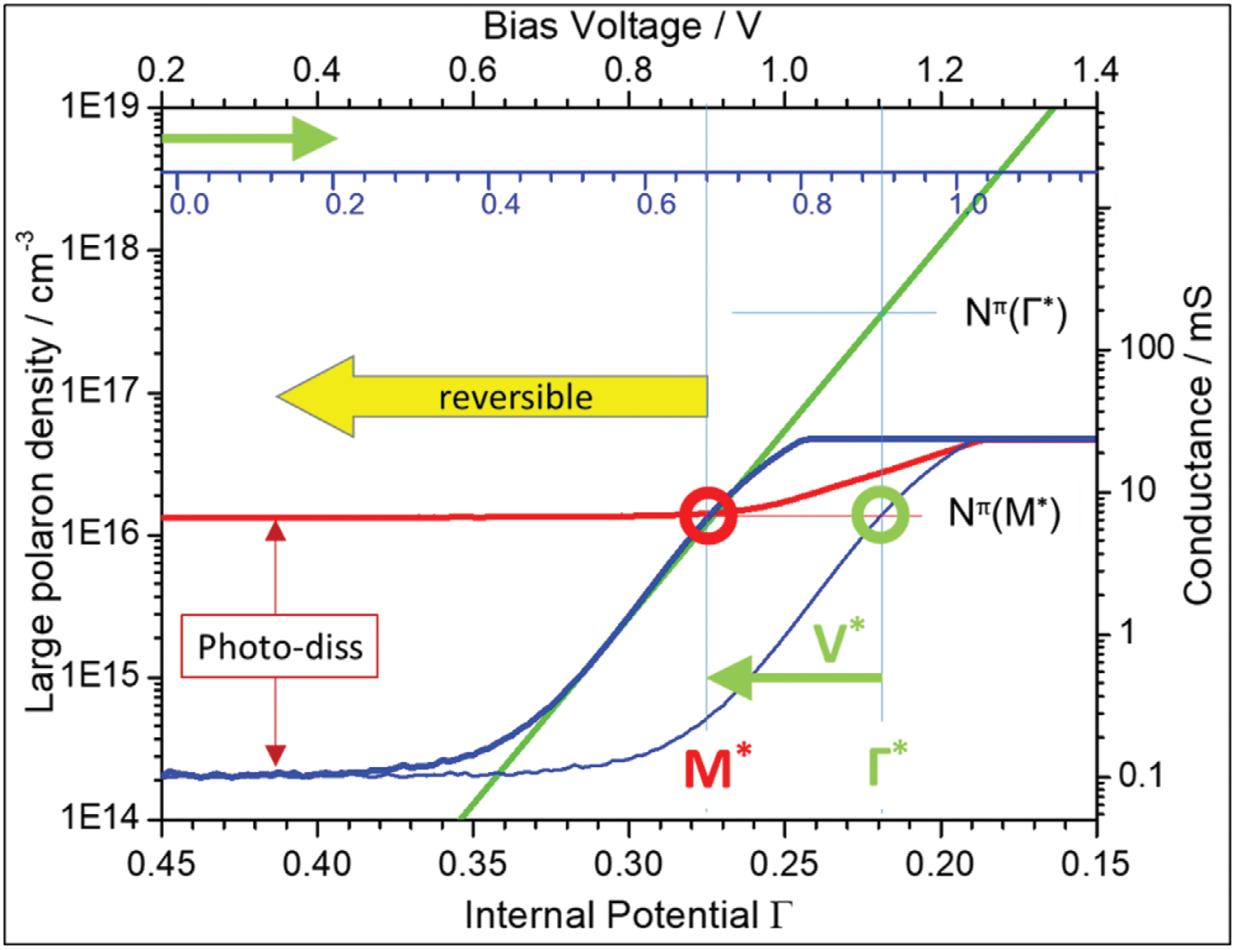
Summary of the FAPI data analyses. Depicted are the critical points of Γ^*^ and M^*^, the dipole V^*^, the as‐measured (thin blue)) and the shifted (bold blue) dark, and the light (red) curves; as well as the N^π^(Γ) dependence (green). The latter one is related to the left axis while all *G–V* data to the right axis. The light, shifted dark, and N^π^(Γ) curves intersect at the M^*^ point. Thereby, the ionicity factor, and the carrier densities of the dark and of the light (N^π^(M^*^)) ohmic regimes as well as of N^π^(Γ^*^) can be derived. The increase in the LaPs density by photo‐dissociation is marked. The yellow arrow visualizes the range (that extends from M^*^ to Γ = 1) of reversible operation within the stability regime of the B^2π^/P^1π^ equilibrium.

In our analysis, the dipole V^*^(V^*^_V_) contributes to Γ^*^+ V^*^ = M^*^, its value V^*^_V_ adds to V_bias_ and it applies for the measured dark curve (thin blue cf., Figure 3a) that refers to the shifted V_bias_ +V^*^_V_ scale (lower voltage axis in **Figure** **5**). This V^*^_V_ contribution needs to be corrected and by transferring the data to the V^*^ = 0 system (upper voltage axis in Figure 5) what is done in Figure 3b (and Figure 5) by the back‐shifted curve (bold blue). In Figure 3b the value of V^*^ = 0.05 (V^*^_V_ = 0.2 V) reduces the idealized (where V^*^ = 0) carrier density from the upper limit N^π^(Γ^*^) = 4⋅10^17^cm^−3^ to N^π^(M^*^) = 1.4⋅10^16^cm^−3^ (where M^*^ = 0.27 (M^*^_V_ = 0.92 V)). This represents the carrier density of the light curve that stays constant with N^π^(M*) while with (the ideal but unrealistic condition) V^*^ = 0 the light curve would be constant with N^π^(Γ^*^) up to the Γ^*^ point.

The voltage separation of dark and light curves by the additional bias component V^*^_V_ (respectively loss dipole V^*^) due to different reference levels is evident only by comparing the dark and the light *G–V* curves and it is completely overseen in the common analyses. In fact, it is the most instructive quantity for judging the performance of the device, to learn about degradation mechanism when its value increases, and to understand the limiting factors for a PSC preparation focusing on a reduction of V^*^ to increase the performance essentially.

In Figure 5 we summarize the main information that are derived by the analyses of the *I–V* FAPI data shown in Figure 2. The figure shows the conductance (right) dependence on the applied bias voltage (top) and the LaPs density (left) as a function of the internal potential Γ (bottom), both in logarithmic scale. The green line gives the exponential relation of N^π^(Γ) [P^1π^] and its slope defines the ionicity factor of the FAPI system (f_i_ = 0.62). It allows the determination of the N^π^(Γ^*^) and N^π^(M^*^) values (marked by thin horizontal lines) and gives the basis for the quantitative description of the gain and loss processes in PSCs. The values of the internal potential at Γ^*^ and M^*^ are indicated by the thin vertical lines, separated by the loss dipole V^*^. The M^*^ point acts as a reference to link the axes of the conductance and of the carrier density. The measured dark curve (thin blue line related to the V_bias_ + V^*^v (blue) scale) is back‐shifted (green arrow) from the Γ^*^ point to the M^*^ point and this curve (thick blue line) refers to the top axis. This back‐shift compensates the V^*^ contribution and now the dark curve, the light curve (red line) and the exponential N^π^(Γ) curve (green) merge at the M^*^ point. The photo‐dissociation occurs by the light dipole V^light^ that dissociates the B^2π^ BiPs and the red double‐sided vertical arrow indicates the additional increase of the P^1π^ carriers to N^π^(M*) = 1.4⋅10^16^cm^−3^. Here, the M^*^ point marks also the upper limit of the stability regime of the B^2π^/P^1π^ equilibrium that guarantees a high reversibility by the pairing and dissociaton equilibrium reaction (as indicated by the yellow arrow).

In Table 1 we list the extracted parameters from the analysis of the dark and light *I–V* characteristics of FAPI solar cells using our novel method. We include the ionicity factor, the deduced internal potential values as well as the calculated (Equation 2) carrier densities.

A correlation of the parameters that can be extracted from our *I–V* characteristics analysis with the efficiency and stability of individual perovskite solar cells requires more sophisticated and deeper analysis and is our ongoing research focus.

The critical values of the intrinsic potentials M^*^ and Γ^*^ and of the dipole V^*^ give a novel meaning to the device performance. Certainly, more data need to be analyzed to obtain their direct relation to the defect chemistry of the active material in the thin film device.

Then, this quantitative analysis of the carrier density taken from experimental *I–V* data enables a better quality control of individually prepared samples and opens new optimization strategies for the perovskite materials. It focusses always on the increase of the N^π^(M^*^) density. This can be achieved by either minimizing Γ^*^ or/and reducing V^*^ for a given iconicity factor f_i_. As an example, in the case of the analyzed FAPI solar cell a reduction of V^*^ by ≈154 mV could cause a ten‐fold increase in N^π^(Γ^*^) and thus in photo‐carrier current density. It is very important to point out that the value of M^*^ separates reversible and non‐reversible contributions to photo‐induced carriers even by the applied V_bias_ during the characterization of PSCs.

Furthermore, the diagram in Figure 5 demonstrates that in solar devices this critical point should never been exceeded to avoid irreversible changes within the charges in the defect sites and formation of additional dipole contributions. A reversible light‐on/off operation can only occur within the regime of V_bias_ ≤ M*_V_. Higher bias voltage values definitely cause damage by irreversible charge‐transfer processes within the IDS and may increase the content of n‐type carriers and the values of either Γ^*^, V^*^, or the number of parasitic dipoles. In fact, this finding is consistent with the frequently reported (and not explained) observation^[^ ^36^ ^]^ that stability in perovskite solar cells is much better when tracking is performed at the maximum power point (MPP at V_MPP_) compared to tracking at increased bias values (V_bias_ > V_MPP_, e.g. at V_OC_) under otherwise identical experimental conditions.

Finally, we comment on the dark curves of the FAPI sample. The frequently reported instabilities of the dark current (also attributed to dynamic properties of perovskite systems) may arise from the voltage stress that appears by applying voltages exceeding the range of reversible reactions. The ohmic regime of the dark curve corresponds to a carrer density of around 2.1⋅10^14^cm^−3^ and this value reflects the large number of parasitic P^1π^ carriers generated without light‐driven dissociation. Rather, they are dissociated from B^2π^ BiPs by structural and electronic imperfections and their number can be reduced by several orders of magnitude. Then, the value of the ohmic section of the dark curve may be used to characterize the sample's stability, providing the use of a sensitive amperemeter. These remarks concern the accuracy of the dark current measurement, the care of avoiding instabilities by parasitic dipoles, and the sample degradation by irreversible processes.

The presented data and their analysis suggest that research effort in materials science could be redistributed and should focus on the reduction of the V^*^ value. Generally, the operation beyond the M^*^ point must be avoided to limit non‐reversible processes within the samples. On the other hand, the size of V^*^ turns out to be the key parameter of degradation studies as most stressed samples exhibit a noticeable increase of V^*^.^[^ ^28^ ^]^

As an outlook, we will try to correlate the changes of the above‐deduced parameters during different degradation processes and to suggest a way for improvement of the perovskite solar cells stability. In this context, we currently investigate the influence of an atomic layer deposited Al_2_O_3_ (ALD‐Al_2_O_3_) film on top of the perovskite film on the values of the critical parameters such as Γ^*^, V^*^, and M^*^. This is to relate these values to the enhanced stability and, in particular, the efficiency of PSCs in a long‐term scale when ALD layers are used as passivation layers in the PSC stack.^[^ ^37^, ^38^ ^]^ We expect further insights into the role of the ultrathin ALD‐Al_2_O_3_ layer on the improvement of the efficiency and long‐term stability of PSCs.

## 5 Summary

Our novel analysis of the *I–V* curves of the FAPI solar absorber is based on the dark curve and its exponential conductance‐bias dependence as it enables a quantitative analysis of the carrier density. Also the value of the ionicity factor f_i_ and the corresponding covalent fraction (1‐f_i_) can be deduced. This analysis reveals that the hybrid perovskite material family is a mixed covalent‐ionic system with covalently bound intrinsic defect states within the ionic gap. These form large p‐type polarons as the main charge carriers that are stabilized by the multi‐atomic nature of their wave functions.

The shape of the dark and the light *I–V* curves reveals the existence of the internal potential values at the Γ^*^ and M^*^ points that appear separated by the loss dipole V^*^. A quantitative analysis is possible and the respective N^π^(Γ^*^) and N^π^(M^*^) values give the basis for the quantitative description of gain and loss processes. The exponential slope of the dark curve ends at the Γ^*^ point, however, it appears to be shifted by V^*^_V_ and for our analysis, it needs to be corrected. Then, it intersects with the light curve at the M^*^ point and N^π^(M^*^) is directly related to the photo‐response. The photo‐dissociation occurs by the light dissociation of the B^2π^ BiPs to p‐type P^1π^ LaPs and the M^*^ point marks the upper limit of the stabillity regime of the B^2π^/P^1π^ equlibrium that guarantees the reversible range of solar cell operation.

The analysis of the dark and light *I–V* characteristics unravels several hitherto unexplained details in the mechanisms of photosensitivity and losses in perovskite materials. With the quantitative analysis of the carrier densities N^π^(Γ) we are able to explain a number of aspects, such as the wide stability range from −2 V up to M^*^, the quantitative assignment of the loss dipole V^*^, and the range of the non‐reversible bias stress. For all these phenomena, the dark curve is an important prerequisite for the analysis of perovskite photo‐sensitive devices.

We are confident that in the future our analysis will reveal novel ways how to produce and operate perovskite solar cells with the highest possible performance and stable device operation.

## Conflict of Interest

The authors declare no conflict of interest.

## Supporting information



Supporting Information

## Data Availability

The data that support the findings of this study are available from the corresponding author upon reasonable request.
