# Supplementary material for: Photosensitivity and Carrier Densities of Perovskite Solar Absorbers
Source: Adv Sci (Weinh). 2025 Feb 25;12(16):2412711. doi: 10.1002/advs.202412711 (PMC12021027; doi:10.1002/advs.202412711)
Supplement: Supplementary file 1 — Supporting Information [file ADVS-12-2412711-s001.pdf]

ADVANCED SCIENCE
Open Access

Supporting Information

for *Adv. Sci.*, DOI 10.1002/advs.202412711

Photosensitivity and Carrier Densities of Perovskite Solar Absorbers

Małgorzata Kot, Katarzyna Gawlińska-Nęcek, Emilia Pożarowska, Karsten Henkel and Dieter Schmeißer**

Supporting Information

Photosensitivity and carrier densities of perovskite solar absorbers

Małgorzata Kot[1,2]*, Katarzyna Gawlińska-Nęcek[3], Emilia Pożarowska[1], Karsten Henkel[1], and Dieter Schmeißer[1]**

[1] Institute of Physics, Brandenburg University of Technology Cottbus-Senftenberg, Konrad-Zuse-Straße 1, 03046 Cottbus, Germany

[2] Faculty of Electronics, Photonics and Microsystems, Wroclaw University of Science and Technology, Janiszewskiego 11/17, Wroclaw 50-372, Poland

[3] Institute of Metallurgy and Materials Science, Polish Academy of Sciences, Reymonta 25 St., 30-059 Krakow, Poland.

Corresponding authors e-mail addresses: *sowinska@b-tu.de; **wdsch13@gmx.de

Preparation of the FAPI device

The FAPI-based perovskite solar cell is prepared on a fluorine doped tin oxide glass washed in water with 2% Hellmanex and isopropanol. Then, the blocking TiO_2 layer was prepared by spin-coating from a precursor based on tetraethyl orthotitanate in ethanol with the addition of HCl. In the following, the thin TiO_2 layer was dried at 200°C for 10 minutes and heat-treated at 500°C for 30 minutes. A framework of mesoporous (mp) titanium oxide was then spin-coated from a precursor based on diluted 30 NR-D paste in ethanol. This film was then dried in 200°C for 10 minutes and annealed at 500°C for 30 minutes. The perovskite precursor solution was prepared in a nitrogen filled glovebox. Lead iodide, formamidinium iodide and methylammonium chloride were mixed in a co-solvent of DMF/DMSO (4:1vol./vol.). The FAPI perovskite layer was deposited using the antisolvent method with diethyl ether. The prepared perovskite precursor solution was spin-coated on the FTO/TiO_2/mp-TiO_2 substrate. Diethyl ether was added on top of FAPI at the 10th second of spin-coating. The layer was then heated at 150°C for 10 minutes to ensure the formation of the black-colored FAPI phase. After cooling, a 40 mM solution of octylammonium iodide in isopropanol was spin-coated onto the perovskite film at a speed of 3000 rpm for 15s, and afterwards the sample was annealed at 100°C for 1 minute. A Spiro-OMeTAD film was used as the hole transport layer. Spiro-OMeTAD was dissolved in chlorobenzene and mixed with a LiTFSI solution in acetonitrile, and 4-tert-butylpyridine was added. The Spiro-OMeTAD solution was spin-coated at a speed of 2000 rpm for 30 seconds. The whole process was completed by the deposition of a gold electrode using physical vapor deposition.

Fig. S1 **The I-V curves of FAPI perovskite solar cells and its parameters**

Intensity:	1000.0	[W/m2]
Isc:	6.553	[mA]
Voc:	1.123	[V]
Imax:	6.077	[mA]
Vmax:	911.371	[mV]
Pmax:	5.539	[mW]
FF:	0.75	[-]
Efficiency:	22.15	[%]
		0.2500
Cell	Area:	00
Rso:	17.788	[Ohm]
Rsho:	25.269	[kOhm]

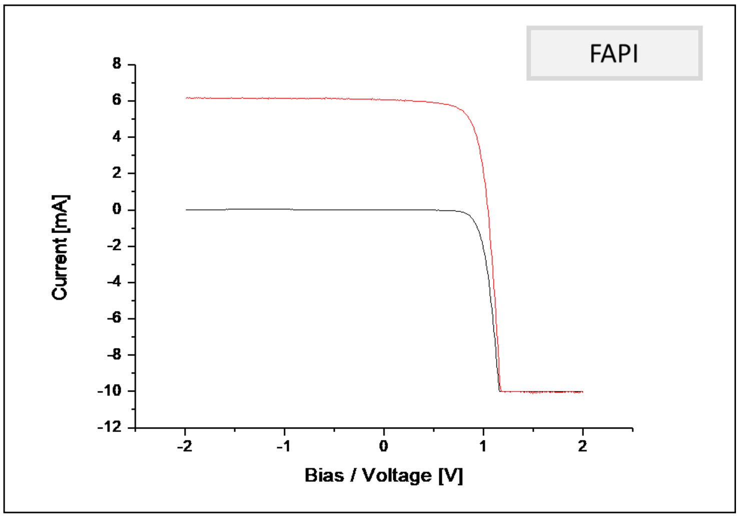

Fig. S2. **Band scheme of a perovskite film extracted from resonant photoelectron spectroscopy map**

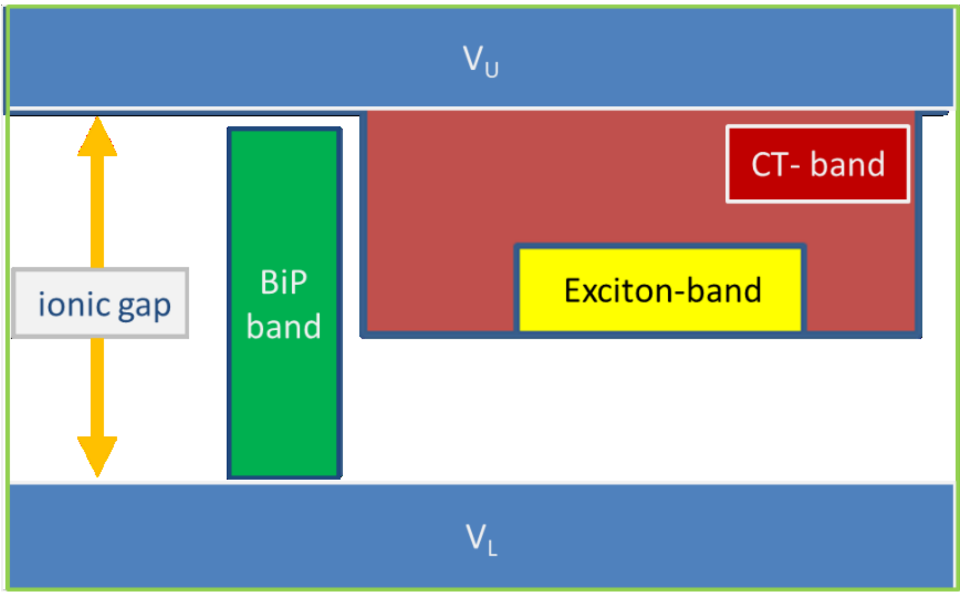

The schematic band scheme of perovskite as derived from the resonant X-ray photoelectron spectroscopy data of methyl ammonium lead triiodide (MAPI) [1]. The ionic gap (orange arrow between the ionization potential V_L and the electron affinity level V_U) and the intrinsic defect states (IDS, orange rectangular) with the charge transfer (CT) band (red) and the exciton band (yellow), and the bipolaron (BiP) band (green) are indicated. The mobile large polarons (LaPs) carriers populate the BiP band.

References

[1] M. Kot, K. Wojciechowski, H. J. Snaith, D. Schmeißer, Chemistry–A European Journal, 2018, 24, 3539-3544.
